# Neurobiological outcomes of cognitive behavioral therapy for obsessive-compulsive disorder: A systematic review

**DOI:** 10.3389/fpsyt.2022.1063116

**Published:** 2022-12-09

**Authors:** Andrea Poli, Andrea Pozza, Graziella Orrù, Ciro Conversano, Rebecca Ciacchini, Daniele Pugi, Nicole Loren Angelo, Lorenzo Lucherini Angeletti, Mario Miccoli, Angelo Gemignani

**Affiliations:** ^1^Department of Clinical and Experimental Medicine, University of Pisa, Pisa, Italy; ^2^Department of Medical Sciences, Surgery, and Neurosciences, University of Siena, Siena, Italy; ^3^Department of Surgical, Medical and Molecular Pathology and Critical Care Medicine, University of Pisa, Pisa, Italy; ^4^Psychiatry Unit, Department of Health Sciences, University of Florence, Florence, Italy

**Keywords:** obsessive-compulsive disorder, functional magnetic resonance imaging (fMRI), magnetic resonance spectroscopy, neuroimaging, Positron emission tomography (PET), cognitive behavior therapy (CBT)

## Abstract

**Introduction:**

Obsessive-compulsive disorder (OCD) is characterized by recurrent distressing thoughts and repetitive behaviors, or mental rituals performed to reduce anxiety. Recent neurobiological techniques have been particularly convincing in suggesting that cortico-striatal-thalamic-cortico (CSTC) circuits, including orbitofrontal cortex (OFC) and striatum regions (caudate nucleus and putamen), are responsible for mediation of OCD symptoms. However, it is still unclear how these regions are affected by OCD treatments in adult patients. To address this yet open question, we conducted a systematic review of all studies examining neurobiological changes before and after first-line psychological OCD treatment, i.e., cognitive-behavioral therapy (CBT).

**Methods:**

Studies were included if they were conducted in adults with OCD and they assessed the neurobiological effects of CBT before and after treatment. Two databases were searched: PsycINFO and PubMed for the time frame up to May 2022.

**Results:**

We obtained 26 pre-post CBT treatment studies performed using different neurobiological techniques, namely functional magnetic resonance imaging (fMRI), Positron emission tomography (PET), regional cerebral blood flow (rCBF), 5-HT concentration, magnetic resonance imaging (MRI), magnetic resonance spectroscopy (MRS), Electroencephalography (EEG). Neurobiological data show the following after CBT intervention: (i) reduced activations in OFC across fMRI, EEG, and rCBF; (ii) decreased activity in striatum regions across fMRI, rCBF, PET, and MRI; (iii) increased activations in cerebellum (CER) across fMRI and MRI; (iv) enhanced neurochemical concentrations in MRS studies in OFC, anterior cingulate cortex (ACC) and striatum regions. Most of these neurobiological changes are also accompanied by an improvement in symptom severity as assessed by a reduction in the Y-BOCS scores.

**Conclusion:**

Cognitive-behavioral therapy seems to be able to restructure, modify, and transform the neurobiological component of OCD, in addition to the clinical symptoms. Nevertheless, further studies are necessary to frame the OCD spectrum in a dimensional way.

## Introduction

Obsessive-compulsive disorder (OCD) is a debilitating psychopathological disorder characterized by the recurrence of repetitive thoughts, urges, or images, which are experienced as intrusive and unwanted (obsessions), and by behaviors that the individual feels the need to perform (compulsions), whose aim is to prevent or reduce anxiety or distress, or prevent some undesired event or situation entailed by the obsession (1). OCD has been shown to be quite common with a 1% prevalence in the general population across many cultures (2). Moreover, OCD often occurs concomitantly with other psychiatric conditions such as anxiety disorders and depression, but among them depression is the most common comorbidity and may constitute a consequence of incapacitating OCD symptoms (3).

In the past few decades, the prevailing models of OCD pathophysiology have focused on cortico-striatal circuitry. A thorough examination of the literature about neurochemistry aspects of OCD is provided in the Supplementary material. At the neurophysiological level, the most consistent result in human OCD studies is the involvement of cortico-striato-thalamo-cortical circuitry (CSTC) (4–6), but other evidence suggests that OCD pathophysiology spans beyond the classic parallel cortico-striatal pathways (7), pointing to a critical involvement in specific roles for the lateral and medial orbitofrontal cortices (OFC), the dorsal anterior cingulate cortex (ACC), and amygdalo-cortical circuitry, suggesting that fear extinction, in addition to behavioral inhibition, is impaired in OCD. Accordingly, neuroimaging studies have highlighted structural and functional alterations in frontal cortical and subcortical regions, which appear to play an important role in the pathogenesis of OCD (8–10). With respect to CSTC circuitry, functioning imaging studies have found orbito-striatal hyperconnectivity in OCD at baseline that normalizes after treatment (4). This highlights how OCD treatments impact not only psychopathological symptoms, but also the neurobiological counterpart. In addition to the pharmacological treatment which resulted to be effective, there is agreement in defining cognitive-behavioral therapy (CBT) as the OCD-specific treatment given its high efficacy rates in decreasing OCD symptoms and increasing patients’ quality of life (11–14).

### Rationale and objectives

Cognitive-behavioral therapy has proved to be effective in the treatment of OCD focusing on patients’ dysfunctional thinking, emotions, and behaviors, and encompasses exposure with response prevention (ERP) and cognitive therapy (15–17). Specifically, the cognitive model suggests that unwanted intrusions (e.g., sexual contact with a relative) are ubiquitous and normal (18, 19), but the interpretation of the intrusion as overly significant, threatening or morally reprehensible differentiates OCD patients from healthy controls (HC) (20). Approximately 70% of OCD patients are responsive to CBT (21) and have lower relapse rates and less adverse side effects than patients who undergo pharmacotherapy (22). ERP was also superior to risperidone and placebo in improving insight, functioning, and quality of life (22). Despite these benefits, harmful residual symptoms, and treatment non-response (nearly one-third of patients with OCD fail to respond to CBT) (21, 23, 24) are common among patients who have undergone CBT, and more research, including psychobiological research, is needed to identify processes that may predict more favorable treatment responses (25). During the past decades, with the improvement of neuroimaging techniques, several studies focused on the biological mechanisms and consequences of CBT in OCD.

No study summarized the available data about the neurobiological changes before and after CBT treatment and the association between neurobiological changes and symptoms improvement in patients with OCD through a systematic review. The aim of this systematic review was to provide an overview of the evidence regarding the neurobiological correlates of CBT-related changes in adult OCD, to discuss the general strengths and limitations and to recommend areas of interest for future research. Specifically, we summarized the available evidence on (i) the neurobiological changes (either structural or functional) after CBT and (ii) the associations between such changes and OCD symptom response after CBT.

## Materials and methods

This systematic review follows the guidelines of Preferred Reporting items for Systematic Reviews and Meta-Analyses (PRISMA) (26, 27).

### Eligibility criteria

Studies were included if: (a) they were conducted in adults (age ≥ 18 years old) with a diagnosis of OCD based upon the criteria of any international classification system [e.g., DSM-5; (1)], (b) they assessed the neurobiological effects of CBT before and after treatment, i.e., pre-post treatment effects, (c) they were based upon any type of research design, (d) they used a single armor compared CBT with any type of control condition (e.g., no treatment or waitlist or another psychological treatment), (e) they were primary studies published in English in peer-review journals before May 2022. Studies were included if they used both outpatients and inpatients. Any formats of CBT (either individual or group), any length of treatment (in weeks) and any type of treatment intensity (frequency of the sessions per week) were allowed. CBT was defined as any psychotherapeutic treatment involving at least one of the CBT active ingredients (i.e., exposure and response prevention and/or cognitive therapy) already found to be effective for OCD in several meta-analyses (17, 25, 28). Concurrent psychiatric medication was not considered as an exclusion criterion since, in routine clinical practice, the majority of patients with OCD receive psychiatric medication (29).

### Search strategy and article selection

Two databases were searched: PsycINFO and PubMed for the time frame up to May 2022. A comprehensive search strategy was elaborated with the help of an experienced librarian. The following keyword “*obsessive-compulsive disorder*” was used and combined through the Boolean operator AND with the keyword “*cognitive-behavioral therapy”* and keywords related to neurobiology (“psychobiol*” OR “neurophysiol*” OR “*neural correlates” OR “psychophysiology” OR “biology”*). In addition, the reference lists of the included studies were examined.

Two authors (CC and LM), working independently, selected the articles at each stage of the review (identification, screening, eligibility, and inclusion) by using Cochrane’s online software for systematic reviews, covidence (30). Authors resolved disagreements through discussion and consensus, and any remaining disagreements were resolved by another author (AG).

### Selection of the studies

**FIGURE 1:**
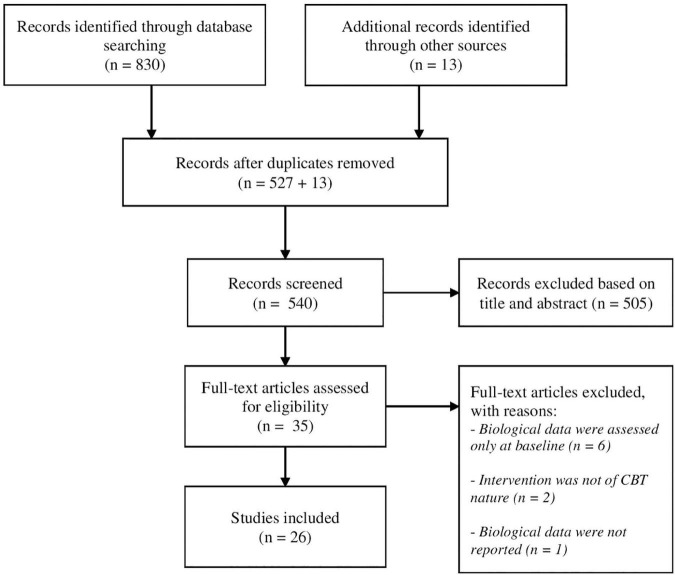
PRISMA flow diagram of search strategy.

The process of selection of the studies is depicted in Figure 1. Database searches allowed to identify 830 articles. The articles were exported to Zotero to eliminate duplicates (*n* = 527). Thirteen additional articles were identified by examination of reference lists of the included studies and other sources. Screening of titles and abstracts led to identification of 35 articles. The main reason for exclusion at the phase of “title/abstract reading” was the investigation of other topics (interventions, electrophysiological studies, critical reviews, etc.). In case there was any doubt, the manuscripts were included for the “full text reading” phase. After the full texts were screened, 26 articles were found to meet the inclusion criteria and nine studies were excluded (see details in Figure 1).

### Characteristics of the studies

Supplementary Table 1 summarizes the papers considered in this review, highlighting design and method of the study, type of CBT treatment, OCD assessment pre-post treatment, and the number of OCD patients included in each of the studies. The included experimental studies showed several different types of methods-paradigms such as: (i) Positron emission tomography (PET) studies (3); (ii) regional cerebral blood flow (rCBF) studies (21); (iii) 5-HT concentration studies (31); (iv) functional resonance magnetic imaging (fMRI) studies (32); (v) magnetic resonance imaging (MRI) studies (1); (vi) magnetic resonance spectroscopy (MRS) studies (21); (vii) electroencephalography (EEG) studies (31).

### Data extraction

Following the PRISMA guidelines, data were extracted in tables independently by three authors (CC, LM, and LA). The methodological quality of the studies was assessed by these authors, working first separately and then together.

We used the National Heart, Lung, and Blood Institute (NHLBI) Quality Assessment Tool for Observational Cohort and Cross-Sectional Studies, which is widely used and recommended by Cochrane for quality assessment of observational and cross-sectional studies (33). The total agreement (Good/Fair/Poor) between assessors was high (20/23 = ∼87%). Inter-rater reliability, measured using Kappa coefficient of Cohen, was high to moderate (*K* = 0.75) (34).

From the selected studies we extrapolated the (i) OCD sample, (ii) sample characteristics (pharmacotherapy, healthy controls, CBT-responders and non-responders), (iii) OCD sample comorbidities, (iv) scales used to assess pre- and post-treatment OCD symptoms, (v) design of the study, (vi) method/paradigm which have been used in the study, (vii) type of CBT treatment and their duration (if reported), and (viii) clinical improvements and, if present, its correlations with post-CBT neurobiological changes.

## Results

### Functional magnetic resonance imaging studies

In the fMRI literature, there are two paradigms mainly used to study brain activations via blood-oxygen-level-dependent (BOLD): (i) resting state fMRI (rsfMRI) and (ii) task-related fMRI.

To reach a better understanding of the brain activity in fMRI of patients with OCD across pre-post treatment we found it necessary to analyze the studies that used these two paradigms separately. Indeed, rsfMRI studies measure the spontaneous brain activity (SBA), i.e., the unsolicited resting activity in the absence of other activities, in which patients are required to reduce all thoughts and relax, whereas task-related fMRI studies show brain activity determined by specific tasks that engage the examined subjects.

#### Resting-state functional magnetic resonance imaging studies

A recent work (35) investigated the coordination of pre- and post-CBT SBA using regional-homogeneity (Re-Ho), a voxel-based measure of brain activity which evaluates the similarity, or synchronization, between the time series of a given voxel and its nearest neighbors. The clinical group was comprised of 22 OCD patients. Each patient had undergone a 12-week CBT treatment, without being administered with a psychopharmacological treatment. The results showed higher Re-Ho values in the medial prefrontal cortex (MPFC), in the cingulate cortex, in the temporal cortex, in the precuneus (PCun) and in the bilateral parietal areas after CBT treatment. Moreover, the study detected lower Re-Ho values in the right orbitofrontal cortex (OFC), in the bilateral dorsolateral prefrontal cortex (DLPFC), in the left cerebellum (CER), in the cerebellar vermis and in the bilateral caudate nucleus. No change was observed in several other areas: right PCun, right caudate nucleus, right calcarine sulcus (CS), right posterior cingulate cortex (PCC), and right medial temporal cortex. The low Re-Ho value found in the caudate nucleus may suggest a lowered ability to process and integrate information from connected brain regions. Conversely, an improvement in the coordination of SBA in the OFC probably underlines a decrease in symptom severity and an enhancement in cognitive functions.

Evidence indicates that the connectivity within cerebral networks in OCD patients seems to change following CBT. Moody et al. (36) investigated changes in brain connectivity of 43 OCD patients after 4-weeks intensive CBT treatment. The results revealed significant increases in connectivity in eight networks. Larger increases involved connections between the CER and the caudate and putamen nuclei, and between the CER and the DLPFC/ventrolateral prefrontal cortex (VLPFC). These results were associated with greater resistance to compulsions.

A recent report (37) highlights that pre-treatment connectivity may reflect an individual’s capacity to recover after intensive CBT, independent of the baseline symptom severity. In particular, pre-treatment FC patterns within the default mode network (DMN) and visual network (VN) significantly predicted post-treatment OCD severity, explaining up to 67% of the variance. These findings have implications for identifying patients that will benefit most from CBT, as well as for understanding the pathophysiology of OCD and its association with CBT symptomatology.

Li et al. (38) investigated whole-brain functional network of 20 drug-naïve and non-comorbid OCD patients performing rsfMRI before and after 12 weeks of CBT. A graph-theory degree centrality (DC) approach and FC method were used to analyze the whole-brain functional network hub and connectivity changes. A significant DC was found in left DLPFC that was reduced after CBT treatment. Resting-state functional connectivity (rsFC) between the left DLPFC and right OFC was increased in the OCD patients at baseline, suggesting emotional salience, and normalized after CBT treatment. RSFC changes between the left DLPFC and DMN positively correlated with changes in clinical symptoms in OCD patients, suggesting increased cognitive control after treatment.

Finally, Rangaprakash et al. (39) estimated the hemodynamic response function (HRF), i.e., the transfer function linking neural activity with the fMRI signal, by performing deconvolution on rsfMRI data from a longitudinal sample of 25 healthy controls scanned twice and 44 adults with OCD before and after 4-weeks of intensive CBT. HRF response height, time-to-peak and full-width at half-maximum (FWHM) in OCD were abnormal before treatment and normalized after treatment in regions including the caudate nucleus. Additionally, pre-treatment response height in caudate nucleus head predicted post-treatment OCD severity (*r* = −0.48, *p* < 0.001), and was associated with treatment-related OCD severity changes (*r* = −0.44, *p* = 0.0028), underscoring its relevance.

#### Task-related functional magnetic resonance imaging studies

Several functional neuroimaging studies have focused on activation changes in frontal cortex following psychotherapeutic and pharmacological treatments for OCD and have shown hyperactivity of the frontal cortex in OCD during task-related fMRI. For this purpose, Nakao et al. (40), using fMRI, investigated cerebral activity in 10 OCD subjects before and after undergoing treatment with fluvoxamine (*n* = 4) or behavioral therapy (BT) (*n* = 6). Each subject was scanned while performing the Stroop test and during a symptom-provocation task. The results showed that, after symptom improvement, administration of the symptom-provoking task revealed significantly decreased activation in OFC, DLPFC, and bilateral ACC in both groups, while activation increased in the parietal cortex and in the CER during the Stroop test.

Nabeyama et al. (41) investigated the alterations of brain function in OCD patients and changes after clinical improvement due solely to 12 weeks-BT. Eleven outpatients with OCD, but not 19 normal controls, received 12 weeks of BT and the authors investigated the differences in the behavioral performance and fMRI results during the Stroop test in patients and normal controls, and their changes after treatment in the patients. The OCD group showed less activation in the ACC and CER with respect to controls. Following significant amelioration in OCD symptoms, the CER and parietal lobe showed increased activation, and the OFC, middle frontal gyrus (MFG), and temporal regions showed decreased activation during the Stroop task, and the task performance improved. The authors’ results suggest that dysfunction of the posterior cerebral regions, in particular the CER, is involved in the pathogenesis of OCD, and that its functional normalization can occur with improvement of OC symptoms.

Freyer et al. (42) used event-related fMRI, to examine 10 OCD patients before and after CBT, in addition to 10 HC, during a probabilistic reversal learning task. The results showed that the reactivity of the OFC and of the right putamen decreased and the activity in the right caudate nucleus increased after CBT.

Baioui et al. (43) studied 12 OCD patients with washing compulsions in a symptom-provocation fMRI experiment. Pictures of standardized and individualized OCD triggers were presented to participants before and after individual twice-weekly 31 CBT ambulatory CBT sessions. With respect to controls, after treatment OCD patients showed reduced activity in important areas of the fronto-striatal network. Individualized symptom provocation was found to promote reductions in nucleus accumbens (NAcc) and posterior supramarginal gyrus (SMG), while standardized symptom provocation promoted reductions in OFC, caudate nucleus, VLPFC, and anterior SMG.

Schiepek et al. (44) studied activation patterns related to the psychotherapeutic process, in order to identify critical instabilities and discontinuous transitions of the change dynamics. Critical phases of the change process were indicated by the maxima of the varying complexity related to the dynamic complexity measure applied. Nine OCD patients with washing compulsions were underwent 8-weeks CBT and were repeatedly scanned (from three to four times) with fMRI during the therapeutic process. Individualized pictures from patients’ personal environments were used for symptom-provoking stimulation. Larger neuronal changes in therapy-relevant brain areas (ACC/supplementary motor area/SMA, bilateral DLPFC, bilateral insula, bilateral parietal cortex, cuneus) were observed during critical phases, suggesting that dynamic changes play a crucial role in the psychotherapeutic process.

Finally, Morgiève et al. (45), in order to study the dynamics of treatment response, evaluated 35 OCD patients, clinically and using fMRI at 4 time points: before, mid-way through, at the end of 3-month CBT treatment and, finally, at 6 month follow-up. The authors used an original exposure task using neutral, generic, and personalized obsession-inducing images. Results showed that clinical improvement was continuous over the course of the intervention and could be predicted by response at mid-therapy. Hemodynamic response to the task was found in the ACC and OFC and was increased during exposure to personalized obsession-inducing images. Furthermore, after exposure to personalized obsession-inducing images, ACC, and the left, but not right, OFC hemodynamic response and anxiety ratings decreased with symptom improvement. Importantly, hemodynamic response continued to decrease with the stabilization of clinical symptoms.

#### Summary of functional magnetic resonance imaging studies

Despite inconsistencies in the results, possibly due to the use of different analysis methods [four studies used whole-brain analysis, two studies employed region of interest (ROI) analysis, one study used graph theoretical analysis, and another study applied a Re-Ho approach], different psychotherapeutic approaches and/or experimental designs, several fMRI studies suggest that the psychotherapeutic treatment of OCD promotes normalization of brain activation patterns. In particular, decreases in activity are reported after treatment in OFC, DLPFC, ACC, and caudate nucleus, and increases have been shown in the parietal cortex and CER. Moreover, reductions in prefrontal regions activity, such as OFC and DLPFC, is more evident in OCD patients who showed a good response to treatment.

### Magnetic resonance imaging studies

Recently, Atmaca et al. (46), compared 12 OCD patients with12 HC using MRI, and found reduced pre-treatment pituitary gland volumes in the OCD group. No change in pituitary gland volume was detected after16-week CBT, suggesting that the clinical efficacy of CBT does not impact on this neural structure in OCD.

Zhong et al. (47) aimed to investigate white matter changes and the effect of CBT on white matter in OCD patients using fractional anisotropy (FA) maps which were acquired using diffusion tensor imaging (DTI). Eighty-five OCD patients underwent DTI before and after 12-weeks CBT sessions, together with 90 healthy controls. The patients group exhibited significantly reduced FA values in bilateral OFC, right CER, and left SPL, while higher FA values were observed in right putamen compared with healthy controls. Following CBT, OCD patients showed higher FA values in right MFG, left OFC, right CER, and left MTG, and decreased FA values in right putamen in comparison with pre-treatment. Furthermore, FA values in the left OFC of patients were significantly positively correlated with the Y-BOCS and its associated “Compulsions” subscale, with the latter being also positively correlated with FA values in the right PUT.

Cao et al. (48) collected diffusion MRI (dMRI) data from 34 unmedicated OCD patients before and after 12 weeks of CBT. 50 healthy controls were also scanned twice at matched intervals. They observed significant group-by-time interactions on the global network clustering coefficient and the nodal clustering of the left lingual gyrus (LG), the left MTG, the left PCun, and the left fusiform gyrus (FFG) of 26 CBT responders in OCD patients. Further analysis revealed that these CBT responders showed prominently higher global and nodal clustering compared to HCs at baseline and reduced to normal levels after CBT. The pre-to-post decreases in nodal clustering of the left LG and the left FFG positively correlated with the improvements in obsessive-compulsive symptoms in the CBT-responding patients.

#### Summary of magnetic resonance imaging studies

Magnetic resonance imaging (MRI) data have been investigated through different methods and paradigms with resulting mixed results. Neuroimaging data highlights a normalization of increased pre-treatment levels especially in subcortical structures, i.e., PUT, and visual regions, such as LG and FFG; whereas other key OCD regions such as OFC and CER show lower pre-treatment FA, which increase after CBT.

### Positron emission tomography studies

Baxter et al. (49) used PET to investigate local cerebral metabolic rates for glucose (LCMRG1c) in nine patients with OCD before and after treatment with either fluoxetine hydrochloride or behavior therapy (BT). After treatment, LCMRG1c in the head of the right caudate nucleus, divided by that in the ipsilateral hemisphere (Cd/hem), was decreased significantly compared with pre-treatment values in responders to both drug and BT. These decreases in responders were also significantly greater than right Cd/hem changes in non-responders and normal controls, in both of whom values did not change from baseline.

Accordingly, using the same sample, Schwartz et al. (50) studied glucose metabolic rates with PET before and after 10 weeks of structured exposure and response prevention (ERP) behavioral and cognitive treatment. Similar to Baxter et al. (49) results, BT responders had significant bilateral decreases in caudate glucose metabolic rates that were greater than those seen in poor responders to treatment. Before treatment, there were significant correlations of brain activity between the orbital gyri with the head of the caudate nucleus and thalamus. These correlations decreased significantly after effective treatment.

In contrast, Apostolova et al. (51), using dynamic PET, showed an increase in glucose metabolism of the right caudate nucleus in 16 patients who responded to both pharmacological treatment and CBT, compared to non-responders. The authors hypothesized that the increase in glucose metabolism of the right caudate nucleus could be associated with the presence of depressive symptoms and/or with the early onset of symptoms in OCD patients of their study.

Lissemore et al. (52) used PET combined with the α-[11C] methyl-L-tryptophan tracer to examine changes in brain regional serotonin synthesis capacity in OCD patients following treatment with CBT or SSRI treatment. 16 patients participated in a 12-week treatment program involving either CBT or sertraline. The study investigated changes in the serotonergic system during OCD treatment. The study highlighted that the SSRI sertraline and CBT significantly both decreased obsessive-compulsive symptoms. This result is associated with a significant pre–post increase in whole-brain 5-HT synthesis capacity in OCD patients who responded to either treatment. Moreover, independent of treatment modality, greater improvement in OCD symptoms with SSRI or CBT was also associated with higher pre-treatment 5-HT synthesis capacity in the raphe nuclei. This work showed the association between higher pre-treatment 5-HT synthesis capacity in the raphe nuclei and greater clinical response to both pharmacological and psychotherapeutic treatments.

Saxena et al. (53), using[^18^F]-fluorodeoxyglucose (FDG) PET, analyzed regional glucose metabolic changes in 10 OCD patients, compared to 12 HC, after a short intensive 4-week CBT treatment. The study highlighted significant bilateral decreases in normalized thalamic metabolism and a significant increase in right DACC activity that was associated with the degree of improvement in OCD symptomatology. Hence, cingulate cortex was proposed as a neural correlate of response to CBT in OCD (54).

#### Summary of positron emission tomography studies

Positron emission tomography (PET) studies which have investigate glucose metabolic changes across time before and after CBT, highlighted the key role of subcortical regions, such as the caudate nucleus and thalamus, which show decreased post-treatment levels. Moreover, DACC have been reported to increase its glucose levels after treatment which correlated with improvements in OCD symptomatology.

### Regional cerebral blood flow studies

Nakatani et al. (55) investigated rCBF, using the Xenon inhalation method, of the basal ganglia in 53 OCD patients treated with BT compared to 31 HC. Prior to treatment, blood flow in the basal ganglia was higher in the patient group. After treatment, however, decreased activity in the right head of the caudate nucleus was shown in patients responsive to therapy (*n* = 22) compared to their pre-treatment value. This decrease tended to correlate with improvement of the functional state.

Yamanishi et al. (56) employed single photon emission computed tomography (SPECT) to investigate the effects of a 12-week CBT in OCD patients resistant to SSRI treatment compared to non-treatment resistant patients. Responders showed decreased metabolism in the left MPFC and in the bilateral MFG (both Brodmann area 10) after treatment. Moreover, the pre-treatment levels of rCBF in the bilateral OFC were significantly correlated with the improvement of symptoms among responders who followed CBT. According to the authors, these results suggest that pre-treatment OFC activation levels may provide predictive information on the subsequent CBT response, while BT may result in changes in rCBF in the medial and middle frontal cortex.

#### Summary of regional cerebral blood flow studies

Neurobiological rCBF data show that an improvement in OCD symptomatology following CBT is accompanied by a decrease in CBF levels in a subcortical region, i.e., caudate nucleus, and prefrontal area, comprising OFC and MPFC. In particular, the pre-treatment activity in the OFC was found to be highly predictive of the response to treatment.

### 5-HT concentration studies

Sampaio et al. (57) investigated whether pre-treatment platelet rich plasma (PRP) 5-HT concentration was associated with latency of treatment response and final response to an ERP protocol for OCD. 30 patients with OCD took part in an 8-week CBT protocol that incorporated 16 ERP sessions. The results showed that in OCD patients with higher baseline PRP 5-HT concentration there was a faster onset of clinical response (at 4 weeks) to ERP. Interestingly, baseline 5-HT concentration was not associated with clinical response at week 8, in spite of sustained clinical improvement of all patients that responded to ERP at week 4.

#### Summary of 5-HT concentration studies

The findings of this study suggest that baseline 5-HT concentration was not correlated with clinical improvement. Nevertheless, higher baseline 5-HT concentration predicts faster clinical response onset (after 4 weeks, rather than 8 weeks) to ERP for OCD symptoms and improvement of depressive symptoms. This is in line with Lissemore et al. (52) results which, using PET to investigate 5-HT concentrations, have reported that higher pre-treatment 5-HT synthesis capacity in the raphe nuclei is correlated to greater clinical response to both pharmacological and psychotherapeutic treatments.

### Magnetic resonance spectroscopy studies

Whiteside et al. (58) used Proton Magnetic Resonance Spectroscopic Imaging (^1^H MRS) to investigate differences in absolute concentrations of neurochemicals (i.e., *N*-acetyl aspartate/NAA) in the head of the caudate nucleus and orbital frontal white matter (OFWM) of 15 adults with OCD before and after twice weekly 16 BT sessions. Results showed that the levels of NAA in the left head of caudate nucleus and of NAA and creatine in the OFWM were significantly lower in patients compared to controls and increased significantly with treatment. This finding suggests the possibility that a successful behavioral treatment may be associated with increases in markers of neuronal viability.

Subsequently, O’Neill et al. (59) investigated neurochemical alterations, induced by a short intensive 4-week CBT treatment, in the cingulate cortex. The glutamate-glutamine cycle (Glu + Gln = Glx, an index of an adequate supply of the neurotransmitter glutamate in the central nervous system) and the NAA-*N*-acetyl-aspartyl-glutamate cycle (NAA + NAAG = tNAA, an index of an adequate supply of the neurotransmitter NAAG in the central nervous system) were examined using MRS. MRS, at clinical field strength, can assay *in vivo* the two most abundant amino acids, Glu and NAA, measured together with the spectrally-overlapping Gln and NAAG, respectively. Previous studies found that OCD patients have shown above-normal pre-treatment glucose metabolism in caudate nucleus, thalamus, OFC and ACC, which are brain structures that form functional neural circuits thought to be hyperactive in OCD (53, 60). Moreover, glucose metabolism has been linked to both Glx and tNAA. Therefore, CBT-induced changes in glucose metabolism may induce or accompany changes in tNAA and/or Glx. Pre-CBT tNAA levels in OCD patients were significantly reduced in the right pregenual ACC (PGACC) but were significantly increased after treatment, reaching HC levels. Pre-CBT Glx levels were significantly increased in OCD patients in the left anterior middle cingulate cortex (aMCC) but were significantly reduced after CBT. The authors also showed that higher pre-CBT levels of tNAA in the right pACC were correlated with a greater pre- to post-CBT decline in OCD symptoms. These results suggest that pACC and aMCC may be cerebral areas responsive to brief intensive CBT treatment in patients with OCD.

Atmaca et al. (61) investigated neurochemical changes in the brain of OCD patients undergoing CBT therapy, by means of magnetic resonance spectroscopy (MRS). In particular, the authors focused on the analysis of the levels of NAA (a marker of neuronal viability) combined with choline (CHO, a marker cell membrane turnover) and creatine (CRE, a marker of cellular energy). Hippocampal NAA/CHO and NAA/CRE ratios were compared in OCD patients and HC, to shed light on the role of neuronal degeneration in the pathophysiology of the OCD. The study involved 12 OCD patients who underwent 16-weeks of CBT and 12 HC. The OCD subjects underwent MRS before and after therapy. Before CBT, OCD patients had significantly lower NAA/CHO ratios, but similar NAA/CRE ratios compared to HC. Pre-to-post-treatment comparisons revealed a significant increase of NAA/CHO ratio levels was found in the OCD group, with respect to HC and post-treatment NAA/CHO ratio levels did not differ between OCD and HC groups. Pre- and post-CBT analyses did not reveal any statistically significant changes in NAA/CRE ratio levels within the OCD group and between the OCD group and HC. Based on these findings, the authors speculated that CBT enhances hippocampal neuronal viability in OCD patients.

#### Summary of magnetic resonance spectroscopy studies

Magnetic resonance spectroscopy studies which have investigated pre-to-post treatment levels of NAA, glucose, CRE, and CHO highlighted that OCD patients are characterized by lower pre-CBT levels of NAA and CRE which increased significantly with treatment, especially in the subcortical caudate nucleus and prefrontal regions, such as OFC, respectively. Moreover, the cingulate cortex seems to play a pivotal role with higher pre-CBT glucose metabolism in the aMCC which decreases after therapy, and lower pre-CBT tNAA levels in PGACC that are reported to increase after treatment in OCD patients.

### Electroencephalography studies

Andreou et al. (62) investigated P300 brain activity patterns in 71 unmedicated OCD patients using a 32-channel EEG during an auditory oddball paradigm. After treatment with sertraline and 10 ± 1 weeks of behavioral therapy, 43 OCD patients underwent a second EEG assessment. At baseline, authors found an increased P300-related activity in particular in the left OFC, but also in left prefrontal, parietal and temporal areas, in OCD patients with respect to control. After treatment, reduction of left middle frontal cortex hyperactivity was found in OCD patients.

#### Summary of electroencephalography studies

The only EEG study with a pre-post treatment design on OCD patients highlights increased pre-CBT P300-related activity especially in OFC. Reduction of pre-treatment hyperactivity in the left middle frontal cortex after treatment, an area associated with uncertainty, might be important for the emergence of OCD symptoms.

## Discussion

We conducted a systematic review of neurobiological changes pre- and post-treatment studies in OCD adult patients. Across the 26 reviewed studies, the total sample undergoing psychotherapeutic treatment was 605 patients with OCD. Among these studies, 15 of them reported a differentiation between responders and non-responders to psychotherapeutic treatment (35–37, 40, 41, 43, 47–51, 53, 56, 57, 63). Generally, this differentiation was determined by setting 45% as the cut-off of the decreases in the total score of the Y-BOCS. By doing so, the samples were divided into CBT-responders (clinical improvements compared to baseline > 45%) and non-responders (clinical improvements compared to baseline < 45%). In total, 384 patients with OCD were analyzed in these 15 studies, of which 298 were reported to be CBT-responders (77.60%) and 86 were CBT non-responders (22.40%), highlighting a high efficacy index of CBT for OCD patients. The effectiveness of CBT lied primarily in the reduction of symptoms severity: in fact, almost the totality of the analyzed studies reported improvements in OCD symptoms after CBT treatment (see Supplementary Table 1). Moreover, research showed that CBT promotes neurobiological changes in the brain of patients with OCD that, in most cases, compensate for the functional and structural changes characteristic of this population. Overall, studies suggested that the psychotherapeutic treatment of OCD leads to the normalization of brain activation patterns especially in CSTC regions. Indeed, among the reviewed studies, OFC and striatum regions, such as caudate nucleus and PUT, were hyperactive in adults with OCD relative to healthy controls and became more active with symptom provocation.

Interestingly, neurobiological data showed the following activity in the above-mentioned regions after CBT treatment: (i) OFC was reported to reduce its enhanced pre-CBT activations in fMRI, EEG, and CBF studies, whereas it showed higher FA values in MRI studies after psychotherapeutic intervention; (ii) striatum regions, i.e., caudate nucleus and putamen, were reported to normalize their abnormal pre-treatment activations across fMRI, CBF, PET, and MRI studies after CBT intervention; (iii) CER demonstrated a reduced pre-CBT activity which increased after treatment in fMRI and MRI studies; (iv) enhanced neurochemical concentrations (NAA, tNAA, and CRE) in MRS studies in OFC, ACC, and striatum regions suggesting a normalized neuronal variability after CBT intervention. In most of the cases, these neurobiological changes were accompanied by an improvement in symptom severity as assessed by a reduction in Y-BOCS scores in OCD patients. Taking everything into account, CBT seems to be able to restructure, modify and transform the neurobiological component of OCD, in addition to the clinical symptoms.

Some limits are present in the current study: our choice of scientific databases, PubMed, and PsycInfo, and the inclusion criteria (such as the English language), could exclude important studies in the field. Another aspect that should be acknowledged concerns the lack of pre-registration of the systematic review protocol. Besides, some of the studies included small sample size that do not allow generalization of the data. Moreover, there were differences among the studies in the type of treatment provided that, although it was CBT, changes in the number and frequency of sessions, and this could have influenced the results. Furthermore, the types of design used varied among the reviewed studies: some of them in fact compared the OCD sample with a group of healthy controls, whereas others compared the neurobiological responses between OCD patients undergoing CBT and drug treatment. In addition, the approach to drug treatment and comorbidities in the OCD sample also differed among the reviewed studies. This resulted in (i) studies that excluded the presence of a current drug treatment during the study, (ii) studies that allowed the OCD patients to continue their current drug treatment, (iii) studies that examined an OCD sample without comorbidities, and iv) studies analyzing OCD patients with different comorbidities. These variables were to be taken into consideration and led to caution in interpreting the results reported above.

Finally, there were differences in outcome measures, although most studies considered percent reduction in the Y-BOCS as the clinical response criteria, the cut-off point for response varied in the different studies. For future research, it might be useful to extend the analysis of the literature on this topic.

Though informative, the prevailing methodological approach in designing neuroanatomical, neurochemical and neurobiological studies investigating CBT effectiveness was that of pooling OCD patient samples. These studies were carried out considering OCD as a unitary syndrome and the total score of OC symptom scales was considered an indication of the disorder severity (35–44, 46–53, 55–59, 61–63). Current OCD conceptualizations (64); deems that this disorder can be better understood as a spectrum of multiple, potentially overlapping, symptom dimensions (namely, contamination, responsibility for harm and mistakes, unacceptable thoughts, and symmetry) and that OCD symptom dimensions have shown to be continuous with normal OC phenomena and occur in OCD patients as well as in the general population.

Assessing potentially overlapping OC symptoms using dimensional measures, such as the Dimensional Obsessive-Compulsive Scale [DOCS; (31)], may therefore be a better approach to understand the relationship between specific OC dimensions and other variables, such as neurobiological correlates. For example, neuroimaging studies previously supported the association between guilt and checking compulsions: Hennig-Fast et al. (65) reported that 20 OCD patients, scanned with fMRI while imagining guilt-inducing scenarios, showed increased responsiveness of the superior frontal, bilateral ACC and superior temporal gyrus. The authors argued that the activations showed by OCD participants during the guilt-inducing scenarios may reflect the neural underpinning of checking symptoms and of modulation of intrusive thoughts in OCD patients, as shown by Mataix-Cols et al. (66). These results suggest the existence of specific neurobiological underpinnings, potentially overlapping with the neurobiological correlates of other OCD dimensions, as previously reported (67, 68). Specifically, biological correlates of CBT intervention should be interpreted along the continuum of each OCD symptom dimension and studies investigating specific OCD symptom dimensions are needed.

## Conclusion

The purpose of the review was to examine all the studies in the OCD literature concerning neurobiological changes before and after CBT treatment in patients with OCD. Neurobiological data from the 26 reviewed studies confirms the brain regions primarily implicated in the pathogenesis of OCD, namely OFC, ACC, caudate nucleus, and CER, and highlights neurobiological changes in the same regions once patients have undergone CBT protocols. CBT appears to normalize increased pre-treatment activity in CTSC regions, and, in most cases, these reductions are accompanied by significant improvements in the scores of the scales used to assess OCD symptomatology (Y-BOCS total scores). Nevertheless, it is considered necessary that future studies help to frame the OCD spectrum in a dimensional way; this may therefore be a better approach to understand the relationship between specific OC dimensions and other variables, such as neurobiological correlates.

## Data availability statement

The original contributions presented in this study are included in the article/Supplementary material, further inquiries can be directed to the corresponding author.

## Author contributions

All authors listed have made a substantial, direct, and intellectual contribution to the work, and approved it for publication.
