# Supplementary material for: Neurobiological outcomes of cognitive behavioral therapy for obsessive-compulsive disorder: A systematic review
Source: Front Psychiatry. 2022 Dec 9;13:1063116. doi: 10.3389/fpsyt.2022.1063116 (PMC9780289; doi:10.3389/fpsyt.2022.1063116)
Supplement: Supplementary file 1 [file Data_Sheet_1.docx]

Supplementary Material

**Supplementary material:** *Neurochemistry of OCD*

1. *Serotonin*

The literature on the neurochemistry of OCD has focused mostly on serotonergic anomalies. It suggests that alterations in serotonin metabolism may be a common biological characteristic of obsessive-compulsive symptoms in OCD as well as in depression (69). Supporting evidence for a role of serotonin in the pathophysiology of OCD is mainly founded on the established therapeutic effects of relatively high doses of serotonin reuptake inhibitors (SSRIs)and the inefficacy of antidepressants with no effect on serotonin transmission or metabolism (20,70-72). Accordingly, pharmacological therapy has been based mostly on the administration of SSRIs, which have the effect of increasing the extracellular levels of this neurotransmitter. However, the serotonergic hypothesis had already been questioned by research on meta-Chlorophenylpiperazine, a serotonergic agonist that increases OCD symptomatology in OCD patients without comorbid depression (18,19) unlike OCD patients with comorbid depression (73). Currently, there is noconsensus on the evidence of significant 5-HT2A receptorchanges in the caudate nucleus and neocortex using PET in unmedicated patients with OCD (74-76). Furthermore, tryptophan depletion has no identifiable effect on symptoms in OCD patients (20,70).

SSRIs treatment may also affect dopamine transmission. PET studies in OCD patients showed increases in striatal D2 receptor availability after repeated SSRI treatment by fluvoxamine, suggesting dopamine downregulation (77). However, SSRIs treatment may also regulate cortical glutamate transmission (78). Overall, it may be important to use a pharmacogenomic approach that would lead to more personalized medicine in OCD (79).

1. *Dopamine*

Evidences supporting a role of dopamine in the pathophysiology of OCD are the beneficial effect of antipsychotic drugs in SSRIs treatment-resistant cases, particularly when tics are present (75,80) consistent with studies where a of loss of D2 receptor availability is reported in the striatum of unmedicated OCD patients, coherent with a relatively increased dopaminergic activity (75,81). Furthermore, dopaminergic mesolimbic hyperactivity may increase the rewarding valence of anxiety-reducing behavioral rituals and confer to compulsions properties akin to those of other “behavioral addictions” (82,83). While extensive evidence exists identifying synaptic concentration of dopamine in the striatum of OCD patients, there is also evidence suggesting the reverse (84), and some neuroleptics, like haloperidol, in some cases can even worsen OCD symptoms (32,85). Overall, an established role for dopamine exists in the pathophysiology of OCD, but its exact role remains to be elucidated.

1. *Glutamate and GABA*

Recent evidence suggests the involvement of other neurotransmitter molecules, beyond serotonin and dopamine in OCD (86,87) showing, for example, that neuronal glutamate transporter EAAC1 limits activation of metabotropic glutamate receptors in the striatum and, accordingly, promotes D1 dopamine receptor expression (88). Previous research pointed out that, in OCD patients, glutamate concentration in the cerebrospinal fluid is enhanced (89). Magnetic resonance spectroscopy (MRS) research highlighted that glutamate levels were increased in the OFC, ACC, and striatum (90,91). Some evidence supports a correlation between striatal glutamate levels and OCD severity (92), and a decrease of glutamate levels after pharmacological treatment or cognitive-behavioral therapy (CBT) (59,93). Hence, several pharmacologic strategies, modulating glutamatergic transmission, have been suggested in OCD, such as memantine, topiramate, and riluzol and lamotrigine (94), but such studies are uncommon and with small samples (89).

GABAergic neurotransmission has been much less investigated in OCD. MRS studies found reduced GABA levels in prefrontal cortex (PFC), including the ACC (95) and an acute increase of GABA levels in MPFC, after successful ketamine treatment, that correlates with OCD improvement (96). Furthermore, a transcranial magnetic stimulation study revealed that the left motor cortex of OCD patients showed shortened cortical silent periods and increased intracortical facilitation, suggesting a reduced GABA_B_ receptor mediated neuronal inhibition that may contribute to the generation and persistence of intrusive thoughts that form the basis for this disorder (97). Overall, these evidences may lead to hypothesize that a reduced activity or number of cortical GABAergic interneurons exists in OCD, and that both directly and indirectly (through ACC to OFC projections), may lead to an aberrant striatal activation. However, a recent MRS study found that, in the ACC, OCD patients displayed a higher estimated GABA level and a higher GABA/glutamate ratio than healthy controls (HC), but no significant group differences were observed in the measure of glutamate (98). These results indicate that ACC GABA abnormalities may be involved in the pathophysiology of OCD. Yet, it seems that glutamate/GABA neurotransmission imbalance may have a particularly important role to play in OCD pathophysiology.
