# Supplementary material for: Neurobiological outcomes of cognitive behavioral therapy for obsessive-compulsive disorder: A systematic review
Source: Front Psychiatry. 2022 Dec 9;13:1063116. doi: 10.3389/fpsyt.2022.1063116 (PMC9780289; doi:10.3389/fpsyt.2022.1063116)
Supplement: Supplementary file 2 [file Table_1.docx]

|  | **Study** | **CBT Sample** | **Sample Characteristics** | **Comorbidities** | **Assessment** | **Design** | **Method - Paradigm** | **Type of treatment** | **Clinical improvements 🡨🡪 Neurobiological changes** |
| --- | --- | --- | --- | --- | --- | --- | --- | --- | --- |
| *Positron Emission Tomography (PET)* | | | | | | | | | |
| **1** | Baxter et al., 1992 | 9 OCD | Free from psychoactive drugs 2 weeks prior to the PET scan.  Confronted with 9 OCD treated with Fluoxetine hydrochloride.  6 CBT-responders, 3 non-responders. | CD, PD, SP | Y-BOCS | Pre-Post | FDG-PET | BT | Y-BOCS scores were decreased post-CBT in responders (from 22.3±4.1 to 13.5±4.0, *p*<.05).  Significant difference in percentage change in right Cd/hem between responders and non-responders (f=3.15, df=7, *p*=.02; Wilcoxon z=2.19, *p*=.03);  positive correlation between percentage change in total Y-BOCS score pre- and post-treatment and the percentage change in right Cd/hem (τ =37, p=.09) |
| **2** | Schwartz et al., 1996 | 9 OCD | Free from psychoactive drugs 2 weeks prior to the PET scan and throughout the study.  6 CBT-responders, 3 non-responders. | CD, PD, SP | Y-BOCS | Pre-Post | FDG-PET | BT | Y-BOCS scores were decreased post-CBTin responders (from 24.3±2.7 to 12.5±2.0, *p*<.05).  Decreased post-CBT Cd/hem (1.27±0.10 vs 1.20±0.12; *t*=3.16, df=17, *p*=.003) with significant difference in percentage change in right Cd/hem between responders and non-responders (Kruskal-Wallis=5.1, *p*=.02);  Positive correlation between percentage change in total Y-BOCS scorepre and post-CBT and the percentage change in right Cd/hem (*τ*=.32, *p*=.07) and left Cd/hem (*τ*=.26, *p*=.l) |
| **3** | Saxena et al., 2009a | 10 OCD | 6 patients were taking SRI medications.  Confronted with 12 HC.  9 CBT-responders, 1 non-responder. | 1 patient with MDD | Y-BOCS | Pre-Post | FDG-PET | Intensive CBT (5 days per week, for 4 weeks) | Y-BOCS scores were significantly decreased post-CBT (from 25.2±3.3 to 11.0±5.1, *p*<.001).  Significant inverse correlation between change in Y-BOCS scores and change in right dACC/Hem values (partial *r* = −0.76, d.f. = 7, *p* = 0.02) |
| **4** | Apostolova et al., 2010 | 9 OCD | Free from psychoactive drugs 3 weeks prior to the PET scan.  Confronted with 7 OCD treated with paroxetine.  6CBT-responders,3 non-responders. | MDD, PD, DD | Y-BOCS | Pre-Post | FDG-PET | 12-weeks CBT | Y-BOCS scores were significantly decreased post-CBT (from 23.2±8.1 to 12.8±8.1, *p*<.001).  Increased in SLMRGlc in right caudate = improvement in Y-BOCS scores.  Kendall *τ* rank of the SLMRGlc in the right caudate was 0.467 and reached a significance level of *p* = 0.060 (n = 10 responders) |
| **5** | Lissemore et al., 2018 | 8 OCD | Free from psychoactive drugs 3 weeks prior to the PET scan.  Confronted with 8 OCD treated with sertraline.  4 CBT-responders, 4 non-responders. | Excluded patients with history of another Axis I disorders | Y-BOCS | Pre-Post | α-[^11^C]methyl-L-tryptophan - PET | 12-weeks CBT | Y-BOCS scores were significantly decreased post-CBT (from 23.0±4.4 to 15.8±7.5, *p*<.001).  Post treatment changes in global K* values (ΔK*_Global_) correlated positively with % decrease in Y-BOCS scores (*r_s_* = 0.46, *p* = 0.08) |
| *regional Cerebral Blood Flow (rCBF)* | | | | | | | | | |
| **6** | Nakatani et al., 2003 | 31 OCD | 21 patients with integrative clomipramine therapy.  Confronted with 31 HC. | No comorbidities | Y-BOCS | Pre-Post | rCBF - Xenon-enhanced CT | 12-weeks CBT | Y-BOCS scores were significantly decreased post-CBT (from 26.77±5.30 to 12.09±4.68, *p*<.001).  No significant correlation between the change of the total Y-BOCS score and the change of the right Cd/hem before and after treatment |
| **7** | Yamanishi et al., 2009 | 45 OCD | Treatment-resistant patientsto a single SRI trial.  33 CBT-responders, 12 non-responders. | Excluded patients with history of another Axis I disorders | Y-BOCS | Pre-Post | rCBF - SPECT using 99mTc-ECD | 12-weeks CBT | Y-BOCS scores were significantly decreased post-CBT (from 33.6±4.5 to 16.2±3.7, *p*<.001).  The reduction in the rCBF in the right OFC was significantly correlated with the reduction in the Y-BOCS (PFDR-CORR b 0.01, corrected) |
| *5-HT concentration studies* | | | | | | | | | |
| **8** | Sampaio et al., 2016 | 27 OCD | Free from psychoactive drugs 3 weeks prior to the scan.  17 CBT-responders, 10 non-responders. | 3 patients with MDD, 2 with SAD, 2 with SP, 1 with BD | Y-BOCS | Pre-Post | PRP 5-HT | 12-weeks CBT | Baseline 5-HT concentration was not correlated with clinical improvement after 8 weeks of ERP (*r* = -0.29; *p* = 0.14), buthigher baseline 5-HT concentration correlated with more pronounced decrease in Y-BOCS scores (percentage change from baseline) after 4 weeks of ERP (*r* = -0.42; *p* = 0.03) |
| *functional Resonance Magnetic Imaging (fMRI)* | | | | | | | | | |
| *Task-related fMRI* | | | | | | | | | |
| **9** | Nakao et al., 2005 | 6 OCD | Free from psychoactive drugs 2 weeks prior to the scan.  Confronted with 4 OCD treated with fluvoxamine and 13 HC.  6CBT-responders. | Excluded patients with history of another Axis I disorders | Y-BOCS  MOCI | Pre-Post | Stroop Task  Symptom-Provocation Task | 12-weeks BT | Y-BOCS and MOCI scores significantly decreased in OCD sample post-CBT (from 29.00±3.59 to 14.60±9.22, *p*<.001; from 16.0±4.03 to 8.90±3.98, *p*<.01, respectively) |
| **10** | Nabeyama et al., 2008 | 11 OCD | Free from psychoactive drugs 2 weeks prior to the scan.  Confronted with 19 HC.  11 CBT-responders. | Excluded patients with history of another Axis I disorders | Y-BOCS  MOCI | Pre-Post | Stroop task  Whole-brain analysis | 12-weeks BT | Y-BOCS scores were significantly decreased post-CBT (from 29.7±3.0 to 12.4±4.9, *p*<.001) |
| **11** | Freyer et al., 2011 | 10 OCD | Free from psychoactive drugs 4 weeks prior to the scan and throughout the study. | 1 patient with history of BN | Y-BOCS | Pre-Post | Probabilistic Reversal Learning Task  Whole-brain analysis | 8-12 weeks CBT | Y-BOCS scores decreased significantly post-CBT (from 25.4 to 14.2, *p*=0.002).  OCD patients with greater reductions of the total Y-BOCS score showed smaller activation increases in the pallidal region (coordinates x, y, z=24, 3, 6; *T*=4.72) |
| **12** | Schiepek et al., 2013 | 9 OCD | 8 patients were drug-free, 1 patient was treated with olanzapine and trazodone before and during the study.  Confronted with 9 HC. | 1 patient with DD | Y-BOCS | Pre-Post | Symptom-Provocation Task | 8 weeks-CBT | Y-BOCS scores were significantly decreased post-CBT (from 26.66±8.62 to 17.33±12.04, *p*<.001).  Brain areas was considerably reduced from pre-therapy (mean of all correlations: *r* = .71, SD = .09) to post-therapy (*r* = .29, SD = .34) with a highly significant difference: *t* (df = 27.0) = 6.10; *p*<.001 |
| **13** | Baioui et al., 2013 | 12 OCD | 5 drug-naïve patients, 2 patients treated with SSRIs.  Confronted with 12 HC.  11 CBT-responders, 1 non-responder. | 1 patient with SP, 1 patient with SAD, 2 patients with MDD | Y-BOCS  OCI-R | Pre-Post | Symptom-Provocation Task  Whole-brain analysis | 31 CBT (Twice-a-week) sessions | Y-BOCS and OCI-Rscores significantly decreased in OCD sample post-CBTfrom 23.08±4.79 to 15.58±7.45, *p*<.001; from 28.08±12.63 to 20.50±12.05, *p*=.005, respectively).  Post-CBT reductions in OFC, caudate and DLPFC (*T*_max_=5.48, p=.033;*T*_max_=4.87, p=.026; *T*_max_=6.89, *p*=.006 respectively) |
| **14** | Morgieve et al., 2014 | 31 OCD | Free from psychoactive drugs 2 weeks prior to the scan.  15 CBT-responders (>45% improvements), 16 low-responders (<45% improvements) | Excluded patients with history of another Axis I disorders | Y-BOCS | Pre-Post + 6 months FUP | Exposure Task (Obsession-inducing Images)  ROI in ACC, OFC, caudate and thalamus | 15-weeks-CBT | The average response over the course of the therapy was 46.3% (S.D. = 23.9%) [Cohen’s *d* = 1.76, 95% confidence interval (CI) 1.17–2.34].  No across-patient significant correlations between changes in left or right OFC nor ACC activations and clinical improvement (all *r^2^*< 0.02, *p* > 0.45) |
| *Resting State fMRI* | | | | | | | | | |
| **15** | Yang et al., 2015 | 22 OCD | Free from psychoactive drugs 4 weeks prior to the scan.  Confronted with 22 HC.  17 CBT-responders, 5 non-responders. | Excluded patients with history of another Axis I disorders | Y-BOCS  OCI-R | Pre-Post | Re-Ho | 12-weeks CBT | Y-BOCS scores were significantly decreased post-CBT (from 24.43±5.99 to 12.33±7.00, *p*<.001).  Post-CBT percentage change in Re-Ho in the right OFC was negatively correlated with the percentage reduction in the Y-BOCS total score (*r* = -0.59, *p* = 0.01) and obsession score (*r* = -0.78, *p* < 0.001). Finally, the percentage change of Re-Ho in the left cerebellum correlated positively with the percentage reductions in the Y-BOCS compulsion score (*r* = 0.63, *p* = 0.006) |
| **16** | Moody et al., 2017 | 43 OCD | 6 patients treated with fluoxetine, 1 with fluvoxamine, 2 with escitalopram, 3 with sertraline and 2 with paroxetine.  Confronted with 24 HC.  42 CBT-responders, 1 non-responder. | 2 PD, 9 GAD, 17 SAD, 7 MDD, 2 DD, 4 BDD, 1 PTSD, 6 SP | Y-BOCS | Pre-Post | rs-fMRI – Whole-brain Network Based Statistic | Intensive CBT (5 days per week, for 4 weeks) | Y-BOCS and OCI-R scores significantly decreased in OCD sample post-CBT (from 24.5±4.7 to 15.0±5.2, *p*<.001; from 1.53±1.0 to 0.92±0.8, *p*<.001, respectively).  No significant associations between changes in Y-BOCS total scores and changes in connection strength in the eight networks that significantly changed pre-to-post-CBT |
| **17** | Reggente et al., 2018 | 42 OCD | 6 treated with fluoxetine, 1 with fluvoxamine, 2 with escitalopram, and 3 with sertraline.  41 CBT-responders, 1 non-responder. | 29 had one or more comorbid psychiatric diagnoses | Y-BOCS | Pre-Post | rs-fMRI –  ROIs Network connectivity | Intensive CBT (5 days per week, for 4 weeks) | Y-BOCS scores were significantly decreased post-CBT (from 24.6±4.7 to 15.0±5.3, *p*<.001).  Post-CBT visual network feature set that also included the amygdala ROIs predict post-CBT Y-BOCS (*R^2^*= 0.24, RMSE = 4.6; *p* <0.001) |
| **18** | Li et al., 2018 | 20 OCD | Drug-free patients.  Confronted with 20 HC. | Excluded patients with history of another Axis I disorders | Y-BOCS | Pre-Post | rs-fMRI - DC and FC  Graph theory | 12 weeks-CBT | Y-BOCS scores were significantly decreased post-CBT (from 23.90±5.62 to 11.95±6.95, *p*<.001).  RSFC changes between the left DLPFC and right PCun positively correlated with percentage reduction changes in the Y-BOCS total score (*r* = .801; *p* < .001) |
| **19** | Rangaprakash et al., 2020 | 44 OCD | Confronted with 25 HC. | - | Y-BOCS | Pre-Post | Rs-fMRI – HRF | Intensive 4 weeks-CBT | Y-BOCS scores were significantly decreased post-CBT (from 24.45±4.66 to 14.93±5.18, *p*<.001).  Significant negative association between percentage change in HRF RH and percentage change in OCD severity (Y-BOCS) with CBT treatment (*R* = -0.44, *R^2^*= 0.19, *p* = 0.0028) in the caudate |
| *Magnetic Resonance Imaging (MRI)* | | | | | | | | | |
| **20** | Atmaca et al., 2018 | 12 OCD | Free from psychoactive drugs 4 weeks prior to the scan.  Confronted with 12 HC. | Excluded patients with history of another Axis I disorders, apart from MDD | Y-BOCS | Pre-Post | MRI - Volume | 16-weeks CBT | Y-BOCS scores were significantly decreased post-CBT (from 22.7±4.3 to 14.4±5.3, *p*<.001).  Pituitary gland volumes did not change significantly after sixteen weeks of treatment (*p*>0.05), with mean values of 0.54±0.29 cm^3^vs. 0.56±0.32 cm^3^(*p*>0.05) |
| **21** | Zhong et al., 2019 | 56 OCD | Free from psychoactive drugs prior to the scan.  Confronted with 90 HC.  38 CBT-responders, 18 non-responders. | Excluded patients with history of another Axis I disorders | Y-BOCS | Pre-Post | MRI – FA | 12 weeks-CBT | Y-BOCS scores were significantly decreased post-CBT (from 23.62±5.73 to 11.36±5.76, *p*<.001).  Post-CBT left MTG FA values significantly positively correlated with the percentage reduction in Y‐BOCS compulsions subscales (*r* = 0.418, *p =* 0.021);  Post-CBT left OFC and right putamen FA values significantly negatively correlated with the percentage reduction in Y‐BOCS obsessions subscales (*r* = −0.373, *p =* 0.019) and Y‐BOCS compulsions subscales (Compulsions subscale; *r* = −0.409, *p* = 0.01), respectively |
| **22** | Cao et al., 2020 | 34 OCD | Free from psychoactive drugs 4 weeks prior to the scan.  Confronted with 50 HC.  26 CBT-responders, 8 non-responders. | Excluded patients with history of another Axis I disorders | Y-BOCS | Pre-Post | Diffusion MRI (dMRI) | 12 weeks-CBT | Y-BOCS scores were significantly decreased post-CBT (from 23.58±6.50 to 8.69±3.82, *p*<.001).  Post-CBT positive correlation (*r* = 0.52, *p* = 0.03) between the decreases in the nodal CP of the lingual gyrus and the reduction rates in the Y-BOCS obsessive subscale.  Post-CBT positive correlation between the decreases in the nodal CP of the FFG and the reduction rates in the Y-BOCS compulsion subscale (*r* = 0.62, *p* = 0.01) |
| *Magnetic Resonance Spectroscopy (MRS)* | | | | | | | | | |
| **23** | Whiteside et al., 2012 | 12 OCD | 8 OCD patients treated with SSRI medications.  Confronted with 15 HC. | Excluded patients with history of another Axis I disorders | Y-BOCS OCI-R | Pre-Post | ^1^H MRSI - NAA-N-acetyl-aspartyl-glutamate cycle Levels | 16 BT(Twice-a-week) sessions | Y-BOCS and OCI-R scores significantly decreased in OCD sample post-CBT (from 24.13±3.2 to 7.64±5.3, *p*<.001; from 27.50±10.3 to 8.82±5.2, *p*=.005, respectively).  Post-CBT Y-BOCS total score was positively associated with change in left caudate Cr (0.73) and mI (0.97), right caudate mI (0.73), and left OFWM cho (0.73) and mI (0.83) |
| **24** | O’Neill et al., 2013 | 10 OCD | 5drug-free patients, 5 with SRIs.  Confronted with 8 HC.  8 CBT-responders, 2 non-responders. | 4 patients with MDD, 1 SP | Y-BOCS | Pre-Post | ^1^H MRSI - Glutamate-glutamine and NAA-N-acetyl-aspartyl-glutamate cycle Levels | Intensive 4 weeks-CBT | Y-BOCS scores were significantly decreased post-CBT (from 28.4±4.4 to 10.8±4.6, *p*<.001).  No significant correlations between post-CBT change in Y-BOCS and post-CBT change in metabolite levels |
| **25** | Atmaca et al., 2015 | 12 OCD | Free from psychoactive drugs 4 weeks prior to the scan.  Confronted with 12 HC. | Excluded patients with history of another Axis I disorders, apart from MDD | Y-BOCS | Pre-Post | NAA/CHO ratio | 16-weeks CBT | Y-BOCS scores were significantly decreased post-CBT (from 22.7±4.3 to 14.4±5.3, *p*<.001). |
| *Electroencephalography (EEG)* | | | | | | | | | |
| **26** | Andreou et al., 2013 | 71 OCD | Co-occurrent sertraline therapy.  Confronted with 71 HC. | Excluded patients with history of another Axis I disorders | Y-BOCS  MOCI | Pre-Post | 32ch-EEG - ERP oddball - P300 | 10 ± 1 weeks BT | Y-BOCS and MOCI scores significantly decreased in OCD sample post-CBT (from 25.13±5.59 to 14.20±7.96, *p*<.001; from 13.51±4.69 to 9.55±6.03, *p*<.01, respectively).  No significant correlations between P300 amplitude or latency with baseline Y-BOCS general score and obsession and compulsion subscores |
